# Neurosurgical Injuries Associated With the Use of Personal Watercrafts

**DOI:** 10.7759/cureus.55664

**Published:** 2024-03-06

**Authors:** Orlando De Jesus, Gisela Murray, Samuel Estronza, Emil A Pastrana

**Affiliations:** 1 Neurosurgery, University of Puerto Rico, Medical Sciences Campus, San Juan, PRI

**Keywords:** spinal trauma, head trauma, injury, jet sky, personal watercraft, neurosurgery

## Abstract

Introduction: A personal watercraft is widely used for recreation on coastlines, rivers, and lakes. This study aimed to identify the spectrum of neurosurgical injuries related to personal watercraft accidents in Puerto Rico.

Methods: A retrospective study was performed utilizing the University of Puerto Rico neurosurgery database to identify patients who had been consulted to the neurosurgery service from 2005 to 2023 due to a personal watercraft-related neurosurgical injury. For each identified patient, basic demographics, injuries received, Glasgow coma scale score at arrival, American Spinal Injury Association impairment scale grade, surgery performed, and outcome upon discharge using the modified Rankin scale (mRS) score were collected. Descriptive statistics were used to report frequency and mean values.

Results: Our service evaluated 11 patients with a personal watercraft-related neurosurgical injury diagnosis during the study period. The mean age of the patients was 35 (± 9). Around 82% of the patients were males. Ejection from the personal watercraft was the mechanism of the trauma in 73% of the patients. Three patients were impacted by a personal watercraft. There were seven spinal injuries and four brain injuries. Among the spinal injured patients, two had neurological deficits. None of the patients with brain trauma required urgent surgery; however, three arrived intubated. Two of them showed signs of diffuse axonal injuries on the head CT scan, while the other had multiple brain contusions. Upon discharge, 70% of the patients had a mRS grade of 0-3.

Conclusions: Personal watercraft accidents causing significant neurological injuries to the brain and spine are infrequent. Injuries were more prevalent among male patients in their thirties. Most patients showed good outcomes when discharged from the hospital. Moderate to severe disability occurred more frequently among spinal injured patients due to residual deficits requiring assistance for ambulation and activities of daily living.

## Introduction

A personal watercraft (PWC), commonly known by its brand names as Jet Ski (Kawasaki Motors, Ltd., Akashi, Japan), WaveRunner (Yamaha Motor Company, Hamamatsu, Japan, or Sea-Doo (Bombardier Recreational Products Inc., Valcourt, Canada), is widely used for recreation on coastlines, rivers, and lakes. Its use has been associated with one-fifth of waterway accidents [1]. Injuries related to its use are uncommon but can cause substantial neurological injuries to the brain and spine, including paralysis and death [2, 3]. In the early 1970s, Kawasaki Motors Corp. U.S.A. introduced the Jet-Ski watercraft, followed by newer models by other manufacturers [4, 5]. However, it was not until 1989 that Vernberg et al. published the first literature report discussing injuries related to their use [6]. The first models could carry one or two people and had 30-40 horsepower motors, allowing speeds up to 40 mph [5, 7]. The technology improved, with current models accommodating three or more passengers, achieving over 340 horsepower and reaching top speeds of 80 mph [4, 7, 8].

Unfortunately, many countries do not require a license or prior certification for PWC usage [9]. In 1995, the estimated injury rate in the United States was 16.2 per 1000 PWC in use [10]. Pikora et al. reported 4.5 accidents per 10,000 hours of use [11, 12]. Regrettably, most accidents are not reported, and adequate epidemiological statistics cannot be produced [5, 13]. In the early days, a significant proportion of the fatalities were attributable to drowning as the PWC operator was not using a personal flotation device [3]. Nowadays, as most individuals wear them, deaths related to drowning are relatively rare compared to those caused by blunt trauma [14].

This study aimed to identify the spectrum of neurosurgical injuries related to PWC accidents in Puerto Rico. It is the first study to examine neurosurgical injuries after PWC accidents specifically.

## Materials and methods

A retrospective study was performed utilizing the University of Puerto Rico neurosurgery database to identify patients who were consulted to the neurosurgery service between 2005 and 2023 due to a PWC-related neurosurgical injury. The neurosurgical database had been prospectively recorded. All the patients were evaluated and managed at a level 1 trauma center. Patients who sustained a neurosurgical injury after a PWC accident were included in the study. Those whose accident did not involve trauma to the head, spine, or peripheral nerves were excluded. Neurosurgical injuries that occurred using another type of watercraft were also excluded. For each identified patient, basic demographics, including age and gender, were collected. In addition, the following variables were investigated: injuries received, Glasgow coma scale score at arrival, American Spinal Injury Association impairment scale (AIS) grade, days of hospitalization, days at intensive care unit (ICU), surgery performed, and outcome upon discharge using the modified Rankin scale (mRS) score. Continuous variables with normal distribution were reported as mean values with standard deviation (SD), while categorical non-continuous variables were presented as numbers with percentages. The University of Puerto Rico Medical Sciences Campus Institutional Review Board reviewed and approved this study. Informed consent was not required because of the study's retrospective nature.

## Results

In a retrospective study performed at our institution, 11 patients with a PWC-related neurosurgical injury diagnosis were evaluated by our service from 2005 to 2023. Approximately 6,500 neurosurgical consults per year were done during the study period. The mean age of the patients was 35 (SD ±9). Around 82% of the patients were males. Ejection from the PWC was the mechanism of the trauma in 73% of the patients. Three patients were impacted by a PWC; one was swimming, another was riding an inflatable water donut pulled by a boat, and the other was riding a PWC impacting another PWC. There were seven spinal injuries and four brain injuries. Patient demographics and characteristics are summarized in Table 1.

**Table 1:** Patient demographics and characteristics SD = standard deviation; PWC = personal watercraft

Variable	n
Age (mean)	35 (SD ±9 )
Male	9 (82%)
Female	2 (18%)
Brain injury	4 (36%)
Spine injury	7 (64%)
Ejected from PWC	8 (73%)
Impacted by PWC	3 (27%)

Among the spinal injured patients, two showed neurological deficits. One patient with a cervical injury had an AIS C grade secondary to cord contusion and nerve root sleeve tears with injury to the brachial plexus. The patient with a lumbar spinal injury had an AIS D grade secondary to an L2 chance fracture accompanied by multiple chest and abdominal injuries. Three of the spinal injuries required surgical treatment with posterior decompression and spinal fusion. Around 57% of the spinal injuries occurred at the thoracolumbar transition zone. None of the patients with brain trauma required urgent surgery; however, three arrived intubated. Two of them showed signs of diffuse axonal injuries on the head CT scan with a GCS of 7 on examination. The other intubated patient had multiple brain contusions on the head CT scan with a GCS of 9. The fourth patient with brain injury had a closed frontal depressed skull fracture and subarachnoid hemorrhage with a GCS of 15. A craniectomy with titanium plate reconstruction was performed a week later to repair the defect. Around 45% of injured patients required admission to the ICU. Three patients with brain trauma and decreased GCS required intracranial pressure monitoring. One patient was transferred intubated to another facility in the United States four days after the accident and was lost to follow-up. Upon discharge, 60% of the patients had a mRS grade of 0-2, 10% had a grade of 3, and 30% had a grade of 4. Among the three patients with a mRS grade of 4, two sustained spinal injuries with residual deficits.

## Discussion

Overall, the most common injuries sustained by patients involved in PWC accidents are fractures to the lower limbs, head traumas with traumatic brain injuries, and spinal injuries [7, 8, 10, 15]. Although head injuries are estimated to account for 14-20% of PWC injuries, they can represent over 50% in the pediatric population [16]. Fortunately, none of the patients in our cohort were children. The population affected is predominantly male, in their twenties or thirties [1, 2, 4, 7, 8, 10, 11, 13, 15, 17]. In our cohort, the mean age of the patients was 35. Most injuries occur secondary to a direct collision with other watercraft or objects, with an additional percent resulting after being forcibly ejected off the watercraft, handlebar injury, axial loading, or hydrostatic jet injury [1-5, 11, 12, 14-18]. In our study, ejection from the PWC was the mechanism of the trauma in 73% of the patients, while 27% were impacted by a PWC. About one-third of injured patients required an ICU admission [1]. In our series, 45% of the patients were admitted to ICU. The overall mortality rate is approximately 8% [1, 4].

Spine fractures or spinal injuries comprise about 18-30% of the injuries [8, 9]. In the current study, spinal injuries occurred more frequently than brain injuries. Donnally et al. noted that lumbar fractures are the most frequent, followed by thoracic fractures [9]. However, other authors reported that the majority of the fractures occurred at the thoracolumbar (T12-L2) transition zone [8, 17]. In our study, 57% of the spinal injuries occurred at the thoracolumbar transition zone. The mechanism for a spinal fracture is usually a hard landing on the seat or top of the PWC after the operator was wave-jumping [8, 17]. Axial loading has been significantly associated with vertebral fractures and spinal injury [1, 4, 9, 17].

Several authors have noted that the most significant contributing factors to accidents are carelessness and inattention, followed by inexperience and excessive speed [2, 3, 5, 7, 8, 18]. Practical education, use of protective equipment, wearing a personal flotation device, and prohibiting minors from using PWC are valuable safety measures. These safety recommendations have been proposed since the first report was published in 1989 [6]. In 2000, the American Academy of Pediatrics Committee on Injury and Poison Prevention published 11 recommendations for the safe operation of PWCs, particularly proposing a 16-year-old minimum age limit for operating a PWC [19]. Additionally, limiting the use of PWCs in swimming areas can reduce injuries caused to swimmers [6, 10]. When used on the coastlines, the PWCs should be operated at a considerable distance from the swimmers on the shoreline.

The current study has limitations. First, the study design was retrospective and observational, which may be subject to bias or errors. Second, the study may have overlooked some cases involved in PWC accidents. Some patients were referred to the neurosurgery service due to a minimal finding on the head or spine CT scan secondary to a PWC injury; however, as no neurosurgical observation was required, they were managed by the trauma service and not documented in the neurosurgery database. Third, this study includes a small sample size for precise analysis due to the reduced number of injuries recorded. Lastly, the study was performed in Puerto Rico, where most accidents occurred along the coastline, which may limit the generalizability of the findings to other countries where PWC usage is more frequent at lakes and rivers.

## Conclusions

PWC accidents causing significant neurological injuries to the brain and spine are infrequent. In this cohort, spinal injuries were more frequent than brain injuries. Injuries were more prevalent among male patients in their thirties. Most patients showed good outcomes when discharged from the hospital. Moderate to severe disability occurred more frequently among spinal injured patients due to residual deficits requiring assistance for ambulation and activities of daily living.
